# Reactive and proactive control processes in voluntary task choice

**DOI:** 10.3758/s13421-023-01470-y

**Published:** 2023-10-05

**Authors:** Victor Mittelstädt, Ian G. Mackenzie, David A. Braun, Catherine M. Arrington

**Affiliations:** 1 Department of Psychology, University of Tübingen, Schleichstraße 4, 72076 Tübingen, Germany; 2 Department of Psychology, Lehigh University, Bethlehem, PA USA

**Keywords:** Cognitive control, Voluntary task switching, Free choice, Proactive control, Reactive control

## Abstract

Deciding which task to perform when multiple tasks are available can be influenced by external influences in the environment. In the present study, we demonstrate that such external biases on task-choice behavior reflect reactive control adjustments instead of a failure in control to internally select a task goal. Specifically, in two experiments we delayed the onset of one of two task stimuli by a short (50 ms), medium (300 ms), or long (1,000 ms) stimulus-onset asynchrony (SOA) within blocks while also varying the relative frequencies of short versus long SOAs across blocks (i.e., short SOA frequent vs. long SOA frequent). Participants’ task choices were increasingly biased towards selecting the task associated with the first stimulus with increasing SOAs. Critically, both experiments also revealed that the short-to-medium SOA bias was larger in blocks with more frequent long SOAs when participants had limited time to prepare for an upcoming trial. When time to select an upcoming task was extended in Experiment 2, this interaction was not significant, suggesting that the extent to which people rely on reactive control adjustments is additionally modulated by proactive control processes. Thus, the present findings also suggest that voluntary task choices are jointly guided by both proactive and reactive processes, which are likely to adjust the relative activation of different task goals in working memory.

## Introduction

Adaptive goal-directed behavior requires cognitive control mechanisms that help to bias information processing towards a currently relevant task goal (e.g., Bugg, 2017; Braver, 2012; Frömer & Shenhav, 2022; Gonthier et al., 2016; Jiang et al., 2018; Verbruggen et al., 2014). Although considerable progress has been made in understanding how people perform tasks according to instructed task goals (forced-choice), less is known about how people decide which task to perform in the first place (free choice; e.g., Brosowsky & Egner, 2021; Brüning et al.; 2020; Dreisbach & Jurczyk, 2021; Fintor et al., 2020; Henare et al., 2020; Imburgio & Orr, 2021; Mittelstädt et al., 2021; Vermeylen et al., 2022; Wong et al., 2022). The present study aims to provide further insights into the mechanisms underlying voluntary behavior by using a novel approach to investigate the possibility that external influences on voluntary task-choice behavior can reflect the strength of reactive control processes that bias task goal activations on-the-fly (i.e., during a trial).

Voluntary task-choice behavior can be investigated by presenting information associated with two independent tasks within a given trial (e.g., Arrington & Logan; 2004; Brosowsky & Egner, 2021; Jurczyk et al., 2019; Mittelstädt, Leuthold et al., 2022; for reviews, see Arrington et al., 2014; Dreisbach & Fröber, 2019). For example, when presented with both a number and a letter, participants can decide whether they want to perform the task associated with the number or letter (e.g., number: odd vs. even, or letter: vowel vs. consonant) with tasks mapped to separate keypress responses (e.g., Arrington, 2008; Mittelstädt et al., 2019). Researchers typically assume that participants maintain two task goals in working memory and that they guide their task choice based on the most active goal representation, which is typically the task most recently performed (e.g., Arrington & Logan, 2005; Braem, 2017; Dreisbach & Fröber, 2019; Orr & Banich, 2014).

Previous studies indicate that participants can engage in proactive regulation of task-goal activations, as task-choice processes can be influenced by contextual factors (e.g., Jurczyk et al., 2021; Mendl & Dreisbach, 2022; Qiao et al., 2022). For example, the strong bias towards selecting task repetitions can be reduced when participants are instructed to select tasks randomly (e.g., Arrington & Reiman, 2015; Liefooghe et al., 2010), or when they learn that task repetitions become increasingly less attractive (cf. Braun & Arrington, 2018; Braem, 2017; Mittelstädt et al., 2018b, 2021). Furthermore, the preference for task repetitions can also be reduced when the proportion of forced-choice trials (only one task stimulus is presented) increases when these trials are intermixed with voluntary choice trials (two task stimuli are presented) within blocks (e.g., Fröber & Dreisbach, 2017; Jurczyk et al., 2021).

Interestingly, several studies have also shown that unpredictable external factors (stimulus characteristics) during a trial influence voluntary task choices (e.g., Arrington, 2008; Arrington et al., 2010; Chen & Hsieh, 2013; Chiu et al., 2020; Mayr & Bell, 2006; Orr & Banich, 2014; Yeung, 2010). For example, participants tend to choose the task associated with stimulus repetitions (e.g., Demanet et al., 2010; Mayr & Bell, 2006) and the task associated with the stimulus that appears first when two task stimuli are presented with randomly varying stimulus-onset asynchronies (SOAs) (e.g., Arrington, 2008; Arrington & Weaver, 2015). Unfortunately, it is difficult to interpret such external effects on voluntary task choices taking place during a trial. On the one hand, these effects could be seen as a failure of control, because they have been primarily observed while participants were instructed to randomly select tasks. Specifically, the randomness instruction requires participants to choose a task goal based on a mental representation of a random sequence and hence not to adapt their choice behavior to external factors (Mittelstädt et al., 2018a). For example, the finding that stimulus repetition effects on task-choice behavior are reduced under low compared to high working-memory load suggests that participants might be simply better at adhering to the instructed goal of selecting tasks randomly under lower load (Demanet et al., 2010). Additionally, the instruction to switch randomly can introduce inhibitory processes that may interfere with the choice processes that typically occur more naturally, making the interpretation of these external influences even more problematic (e.g., Liefooghe et al., 2010; Lien & Ruthruff, 2008; Mittelstädt et al., 2018a; Mittelstädt et al., 2023).

On the other hand, external influences that influence voluntary task choices might reflect reactive control processes rather than a lack of control. Some recent studies have observed that voluntary task choices are also biased by external factors when participants are not instructed to randomly select task (e.g., Chiu et al., 2020; Mittelstädt et al., 2022). For example, participants adapted their task behavior to unpredictable changes in the perceptual discriminability of stimuli, which indicates that they were able to flexibly adjust task-goal activation, and thus task choices, on-the-fly towards a less effortful task (Mittelstädt et al., 2022). However, one might argue that such external influences on choice behavior are not truly under participants' control, and instead reflect unintentional passive processes that bias the online activation of task goals. To provide a more diagnostic test of reactive control on voluntary task choices, it is necessary to demonstrate that people can regulate the extent to which external factors influence behavior.

### The present study

The present study addresses this issue by investigating whether the availability of task information more strongly biases voluntary task choices in conditions when globally learned expectancies about stimulus availability favor such externally driven task-choice behavior. Thus, our primary research question was to see whether people are able to control the degree to which changes of task-specific information can bias their task choices during a trial. For this purpose, we conducted two preregistered experiments, where participants could select which of two tasks (i.e., letter and number task) to perform in a trial without any additional instructions. We manipulated the stimulus availability of the two tasks in a way that the stimulus of one of the two tasks (i.e., letter or number) was displayed immediately following an intertrial interval, whereas the stimulus of the other task appeared after an SOA of 50 (short), 300 (medium) or 1,000 ms (long). Critically, participants could not predict which task would be presented first, and hence participants were only able to adapt their task-choice behavior to the specific SOA during a trial and not in advance. Importantly, we also varied the relative frequencies of short (50 ms) compared to long (1,000 ms) SOA, such that in half of the blocks there was a high (low) proportion of short (long) SOAs and vice versa.

In general, we assume that people are more likely to choose the task associated with the first presented stimulus when the delay (SOA) of presenting the second stimulus increases from short to long, as has been observed in voluntary task-switching (VTS) studies with randomness instruction (e.g., Arrington, 2008). This main effect of the trial-specific SOA suggests that task-choice behavior can be influenced by fluctuations in task availabilities, even without explicit instructions to choose randomly. However, these external influences on task-choice behavior occurring during a trial do not provide conclusive evidence to differentiate between accounts that explain externally driven task-choice behavior as being under control or as a failure of control. Thus, the main question we aim to address is whether people’s sensitivity to the trial-wise varying SOAs (as reflected in a bias to select the task associated with the first stimulus (S1-task percentages as dependent variable)) across trials is larger in the blocks with a high frequency of long SOAs versus blocks with a high frequency of short SOAs. If so, it would demonstrate that participants intentionally use a control strategy to modulate the extent to which external factors bias their task-choice behavior based on global expectations. Since the decision to select the first available task over the second is made within a trial, we interpret the modulation effect of the trial-specific SOA on S1 percentage by the block-wise frequency-SOA condition (i.e., short or long SOA frequent in a block) as indicative of a *reactive* control strategy.

Additionally, Experiment 2 explores how engaging in preparatory task-choice processes (i.e., proactive control in advance of a trial) affects the external influences of local and global SOA manipulations on choice behavior by varying the intertrial interval (ITI) between trials. Previous studies employing the VTS paradigm with randomness instructions have suggested that participants utilize the time preceding a trial to initiate task selection, potentially making their decision before any task-specific information is presented, particularly with longer intervals (Arrington, 2008; Arrington & Logan, 2005). Therefore, we assume that individuals will also engage in proactive task selection in the present VTS environment, even without explicit randomness instructions, particularly when provided with a longer ITI. If so, an interaction between trial-specific SOA and frequency-SOA condition on S1-task percentages observed in Experiment 1 might be more pronounced (or exclusively present) in trials with short ITIs. This finding would indicate a dynamic interplay between proactive and reactive processes in guiding voluntary task choices.

## Experiment 1

In this experiment, participants freely choose between two tasks (letter and number) in each trial. We manipulated the availability of the task stimuli by presenting one task's stimulus first, followed by the stimulus of the other task after a trial-specific SOA (50 ms, 300 ms, or 1,000 ms). Additionally, we varied the relative frequencies of short and long SOAs within blocks (short SOA frequent (SF) or long SOA frequent (LF), cf. Table 1). Our primary focus was to examine whether a stronger preference for selecting the task associated with the first stimulus (S1 percentage) with longer trial-specific SOAs is more pronounced in LF blocks compared to SF blocks. If this is the case, it would suggest that external influences on task-choice behavior within a trial can be adjusted based on a reactive control strategy.

**Table 1:** Number of trials per block at each stimulus onset asynchrony vs. number-letter) were equally distributed across trials

		Trial-specific SOA		
		Short (50 ms)	Medium (300 ms)	Long (1,000 ms)
Frequency-SOA condition	Short SOAs frequent (SF)	128	36	36
	Long SOAs frequent (LF)	36	36	128

### Method

#### Participants

As preregistered,^1^ 40 participants were tested online. All participants provided informed consent and could receive course credits for their participation. Data from ten participants were excluded due to excessively high repetition rates in correct trials after our data preparation procedure (> 95% task repetitions, cf. preregistration).^2^ Moreover, one additional participant was excluded due to accuracy below 80% (i.e., 38% correct trials). Note that the results for task-choice behavior were very similar when including all participants (including the significant interaction between trial-specific SOA and frequency-SOA condition although with a reduced effect size). The remaining 29 participants (18 female, 25 right-handed) ranged in age from 18 to 32 years (M = 21.59 years).

#### Apparatus and stimuli

The experiment was conducted online using the JavaScript library jsPsych (De Leeuw, 2015). All visual stimuli were presented in black on a grey background. A centrally positioned plus sign served as fixation point. Stimuli were the numbers 1, 2, 3, 4, 6, 7, 8, and 9 and the uppercase letters A, B, C, D, W, X, Y, and Z. For the number task, participants had to press a left (right) key when the number was smaller (larger) than 5. For the letter task, participants had to press a left (right) key when the letter was before (after) M in the alphabet. The stimuli of the two tasks appeared one above the other at the center of the screen. Following Fröber and Dreisbach (2017), the task-specific stimuli positions were kept constant throughout the experiment but counterbalanced across participants. For half of the participants, the letter appeared at the top, whereas for the other half of participants this mapping was reversed. Task stimuli at the top were always answered with key presses (“Q” and “W”) made with the index and middle fingers of the left hand and task stimuli at the bottom were always answered with key presses (“O” and “P”) made with the index and middle fingers of the right hand.

#### Procedure

All participants were instructed that stimuli appeared at different times, and they were free to perform whichever task they wanted. Half of the participants started with blocks with a high frequency of long SOAs (i.e., condition LF) followed by blocks with a high frequency of short SOAs (i.e., condition SF), whereas this order was reversed for the other half of participants. In each block type condition, participants first performed one practice block with 50 trials followed by two experimental blocks with 200 trials each. As can be seen in Table 1, each block of the LF condition consisted of 64% of trials with a long SOA (1,000 ms) and with the other trials equally distributed over the short SOA (50 ms) and medium SOA (300 ms). The percentages were reversed for the SF condition – that is, there were 64% of trials with short SOA and the other SOAs occurred in 18% each. The specific stimulus orders (i.e., letter-number

At the beginning of each trial, the fixation cross appeared on the screen for 500 ms. The first stimulus (letter or number) was displayed immediately at the offset of the fixation cross and the second stimulus was added to the display at the end of that trial’s SOA. The specific identities of stimuli in each trial were selected randomly with the constraints that no stimulus presented on the current trial was presented in the previous trial. The stimulus (stimuli) remained on the screen until the participant responded or up to a response deadline of 3 s. Following correct responses, a screen with the fixation cross with an intertrial interval (ITI) of 500 ms was presented before the next trial started. In case of an error (or no response within the response deadline), an additional error screen was presented for 3 s (first practice block: 4 s) indicating that an error has been made (or that the response was too slow) and providing the S-R mappings for the two tasks.

#### Data preparation

The practice blocks and the first trial of each block were excluded from any analyses. We then categorized the task performed on each trial based on the hand used to respond. We excluded trials with response times (RTs) less than 200 ms (1.8%; including trials in which a response was given prior to onset of the stimulus related to the performed task), trials without any response within the response windows (0.7%), error trials (5.8%), and trials following incorrect responses (6.5%). In total, 88.0% of the experimental trials were included in the analyses.

#### Design

We investigated the percentage of trials in which participants selected the task related to the first-presented stimulus as a function of the experimental conditions (i.e., trial-specific SOA and frequency-SOA condition). Specifically, we conducted a repeated-measures ANOVA with the within-subject factors of trial-specific SOA (short, medium, long), and frequency-SOA condition (SF, LF) on the S1-task percentages.^3^ Assuming that there is a larger bias of S1-task percentages when the SOA in a specific trial is longer (i.e., main effect of trial-specific SOA), we are particularly interested in whether this effect is further modulated by frequency-SOA condition (i.e., interaction of trial-specific SOA and frequency-SOA condition). Specifically, a stronger increase of S1-task percentages with a longer SOA in LF than SF blocks would demonstrate that participants can control the strength of external influences on their voluntary task-choice behavior. It should be emphasized that the analyses reported in the main texts does not consider whether S1 was associated with repeating or switching tasks. In additional (not preregistered) analyses, we also checked the effects on S1-task percentages separately for whether S1 was associated with switching or repeating tasks (see Appendix B).

### Results and discussion

**Fig. 1:**
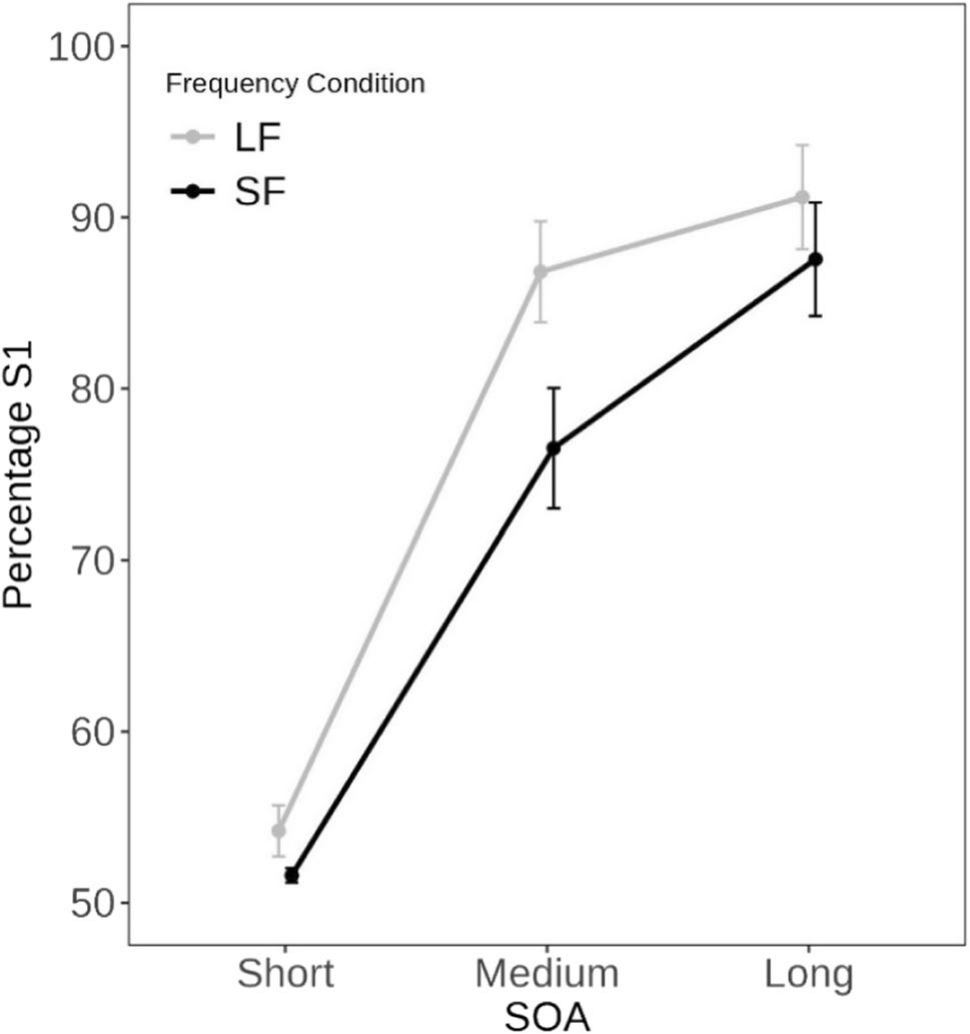
Percentage of trials in which participants selected the task related to the first-presented stimulus (S1) as a function of the trial-specific stimulus-onset asynchrony (SOA) in trial n and the frequency of SOA in this block (short SOA frequent (SF), long SOA frequent (LF)). Note that the order of stimuli in each trial was randomly selected. Standard error of the means is given in parentheses

Figure 1 shows the percentage of S1-task choices as a function of trial-specific SOA and frequency-SOA conditions. The 3 × 2 ANOVA revealed a main effect of trial-specific SOA, *F*(2, 56) = 140.40, *p* < .001, η_p_^2^ = .83, indicating that participants were more likely to select the task associated with S1 as the trial-specific SOA increases (short: 53.0%, medium: 82.8%, long: 90.6%, with all pairwise comparison *p*s < .001). A significant main effect of frequency-SOA condition reflected a lower percentage of S1 responses in blocks with frequent short SOAs compared to frequent long SOAs (72.6% vs. 78.4% in SF and LF blocks, respectively), *F*(1, 28) = 19.06, *p* < .001, η_p_^2^ = .41. There was also a significant interaction between the two factors, *F*(2, 56) = 6.69, *p* = .002, η_p_^2^ = .19.^4^ As can be seen in Fig. 1, the difference between short and medium trial-specific SOAs was larger in the LF (Δ = 33.8%) than the SF blocks (Δ = 25.9%), suggesting that participants were more sensitive to the increase in trial-specific SOAs in blocks in which long SOAs were more frequent. Note that the difference between medium and long SOAs was smaller in the LF (Δ = 4.4%) than the SF condition (Δ = 11.1%) – presumably because the bias to select the task associated with S1 was already at ceiling (cf. Loftus, 1978, for interpreting interactions with percentages in light of ceiling effects).

Thus, both the trial-specific SOA and the block-wise frequency-SOA manipulations significantly influenced voluntary task-choice behavior, and importantly, interacted with each other in determining task choice. The trial-specific SOA effect reveals that participants exhibit a bias in their task-choice behavior within a trial, whereby they respond to the first stimulus and display an increasing tendency to select the task associated with S1 as the duration of S1 prior to the onset of S2 extends (for a similar finding using the VTS with randomness instruction, see, e.g., Arrington, 2008). The interaction between the trial-specific SOA effect and frequency-SOA indicates that participants exhibit a stronger reliance on trial-specific changes in task-specific information availabilities when the overall task environments strongly support such behavior. This finding suggests that participants employ a reactive control strategy to adjust the degree to which they rely on external factors in guiding their task choices.

As can be seen in Appendix C, similar interactions of block-wise frequency-SOA and trial-specific SOA on S1-task percentages were present when additionally considering whether S1 was associated with switching or repeating tasks. Interestingly, however, the interactive effects were stronger for potential switch-S1 than repetition-S1. In fact, for repetition-S1, the pattern was only descriptively similar, but the interaction was not significant. Thus, this seems to suggest that participants particularly relied on reactive control strategies to facilitate flexible behavior (i.e., task switching). However, it is important to exercise caution when interpreting these findings, as there is a need to consider the substantial bias towards selecting when this stimulus is associated with repetition (> 90% across conditions). Since the specific conditions have already approached the maximum value (i.e., 100%), this leads to limited variability and reduced sensitivity in detecting potential interactions (see Loftus, 1978).

## Experiment 2

The most important finding of Experiment 1 was that participants were more strongly influenced by the short-to-medium trial-specific SOA increase in the LF than SF condition suggesting that participants more strongly reactively biased their task choices based on the trial-specific SOA during a trial when it was more favorable to do so. Experiment 2 was designed to investigate how varying the interval between trials (i.e., short vs. long intertrial interval [ITI]) affects the stability of the SOA manipulation effect on task-choice behavior observed in Experiment 1. Previous studies using the VTS paradigm with randomness instructions have demonstrated that stimulus availability effects on task-choice behavior are reduced with long compared to short ITIs (e.g., Arrington, 2008). This finding suggests that participants are better able to randomly select a task in advance of a trial when they have more preparation time, which in turn makes them less susceptible to external influences during the trial. Thus, varying the ITI alters the amount of time that participants are able to engage in proactive control associated with task choice processes just prior to stimulus onset. Thus, unlike the block-wise manipulation of frequency-SOA in Experiment 2, which provided information about the overall pattern of reactive control across the experiment, the manipulation of ITI directly influences the degree of proactive control in guiding voluntary task choice. Therefore, if participants' inclination towards more proactive task-choice behavior with a long ITI compared to a short ITI further influences the interaction between trial-specific SOA and block-wise frequency-SOA manipulations on choice behavior, it would suggest that task-choice behavior embodies a dynamic interplay between reactive and proactive control processes.

### Method

The method, apparatus, stimuli, and procedure were similar to those in Experiment 1 except for the following changes: The ITI varied randomly across trials (short = 300 ms; long = 1,100 ms). Because of this additional factor, we decided to increase the sample size to 60 participants.^5^ Following the same data-preparation procedure as in Experiment 1, the data of eight participants were excluded due to excessive use of global task-selection strategies (e.g., always selecting the letter task throughout the experiment). The remaining 52 participants (45 female, 46 right-handed) ranged in age from 18 to 46 years (M = 21.06 years). As in Experiment 1, the results for task-choice behavior were again very similar when including all participants (including the significant interaction between trial-specific SOA and frequency-SOA although with a reduced effect size).

#### Data preparation

We followed the same data-preparation procedure. Thus***,*** the practice blocks and the first trial of each block were excluded from any analyses. We excluded trials with RTs less than 200 ms (1.0%; including trials in which a response was given prior to onset of the stimulus related to the performed task), trials without any response within the response windows (0.1%), error trials (5.6%) and trials following incorrect responses (5.7%). In total, 89.1% of the experimental trials were included in the analyses.

#### Design

As mentioned in Experiment 1, potential ceiling effects in the trial-specific SOA condition with long SOAs make the interpretation of results including this condition difficult. Thus, as we also mentioned in our preregistered document, we first conducted a repeated-measures 2 × 2 × 2 ANOVA with the within-subject factors of trial-specific SOA (short, medium), frequency-SOA condition (SF, LF), and ITI (short-ITI, long-ITI) on the S1-task percentages. In the analyses reported in Appendix B, we additionally consider whether S1 was associated with switching or repeating tasks. In Appendix C, we also report additional analyses including the trial-specific long SOA condition.

### Results and discussion

**Fig. 2:**
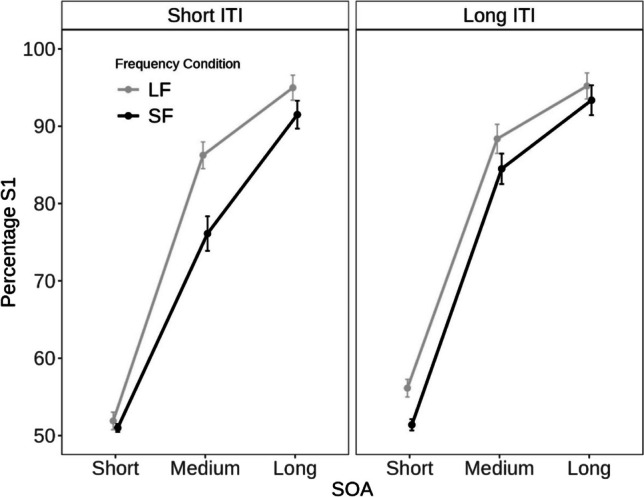
Percentage of trials in which participants selected the task related to the first-presented stimulus (S1) as a function of the trial-specific stimulus-onset asynchrony (SOA) and intertrial interval (ITI) in trial n and the frequency of SOAs in this block (short SOA frequent (SF), long SOA frequent (LF))

Figure 2 shows the percentage of trials in which participants selected the task related to the first-presented stimulus separately for each condition.

The 2 × 2 × 2 ANOVA with the within-subject factor of trial-specific SOA (short, medium), frequency-SOA condition (SF, LF), and ITI (short-ITI, long-ITI) on the S1-task percentages revealed that all main effects were significant: The main effect of trial-specific SOA indicated that participants were more likely to select the task associated with S1 at medium (83.8%) than at short SOA (52.6%), *F*(1, 51) = 340.32, *p* < .001, η_p_^2^ = .87. The main effect of frequency-SOA condition reflected a lower percentage of S1 choices in SF (65.7%) compared to LF blocks (70.7%), *F*(1, 51) = 32.32, *p* < .001, η_p_^2^ = .39. The main effect of ITI indicated a smaller bias to select the task associated with S1 at short-ITI (66.3%) than at long-ITI (70.1%), *F*(1, 51) = 23.98, *p* < .001, η_p_^2^ = .32, conditions. The two-way interaction between SOA and ITI was also significant, *F*(1, 51) = 6.55, *p* = .014, η_p_^2^ = .11, reflecting larger differences on S1-task percentages between short and medium trial-specific SOAs with long-ITI (SOA_medium_ = 86.4%; SOA_short_ = 53.7%) than with short-ITI (SOA_medium_ = 81.1%; SOA_short_ = 51.5%) conditions. Critically, the two-way interaction between SOA and frequency-SOA condition was also significant, *F*(1, 51) = 6.79, *p* = .012, η_p_^2^ = .12. As in Experiment 1, there was a larger difference on S1-task percentages between short and medium trial-specific SOAs in LF (87.3–54.0%) than SF blocks (80.3–51.2%). Thus, this interaction once again suggests that participants employ a reactive control strategy to regulate the influence of task-specific information on their task choices.

Interestingly, the three-way interaction was also significant, *F*(1, 51) = 13.16, *p* = .001, η_p_^2^ = .21. Separate 2 × 2 ANOVAs were conducted for each ITI condition. For the short-ITI condition, the two main effects as well as the interaction was significant (all *p*s < .001 and all η_p_^2^ > .24). For the long-ITI condition, the two main effects (all *p*s < .001 and all η_p_^2^ > .26) but not the interaction (*p* = .671 and η_p_^2^ < .01) were significant. As depicted in Fig. 2, this three-way interaction indicates that the differences between short and medium trial-specific SOA were exclusively modulated by the frequency-SOA condition with a short ITI and not with a long ITI. In other words, it appears that participants predominantly relied on a reactive control strategy when they had limited time to proactively select a task. Thus, the three-way interaction highlights the dynamic interplay between proactive and reactive control in task choice. Specifically, the degree of proactive control engaged by participants appears to regulate the extent to which they rely on environmental influences.

The additional analyses in Appendix B demonstrate the stability of the present findings by showing that the interaction between frequency-SOA condition and trial-specific SOA (i.e., collapsed across ITI) was present for both potential switch-S1 and repetition-S1. Note, however, only for potential switch-S1, not repetition-S1, was this interaction restricted to the short ITI. It seems possible that due to a strong task-repetition bias (i.e., overall strong bias to select repetition-S1), the resulting ceiling effects make it difficult to detect a potential modulation of ITI.

## General discussion

The present study investigated whether and how people incorporate external influences into their voluntary task-choice behavior. The results of two experiments indicate that participants biased their task-choice behavior during a trial towards the first of two presented task stimuli separated by a variable trial-specific SOA (short, medium, long). Critically, in both experiments, participants were more strongly biased by the short-to-medium trial-specific SOA increase when they were expecting long SOAs in the LF frequency-SOA condition compared to the SF frequency-SOA condition when they had limited time to prepare for an upcoming trial. This suggests that people have the ability to adjust the strength of external influences on task-choice behavior. As participants were unable to predict which of the two task stimuli would be presented first, we interpret this interaction as indicative of reactive control adjustments, wherein participants regulate external influences to guide their voluntary task choices. In Experiment 2, the interaction of trial-specific SOA and frequency-SOA was reduced (and not significant) with increased preparatory processes in advance of trial (i.e., with longer ITIs). This suggests that the amount of time allowed to exert proactive control can also reduce the degree to which participants engage in reactive control.

In general, current theoretical accounts assume that that people choose a task once it is sufficiently activated (e.g., Chiu et al., 2020; Dreisbach & Fröber, 2019; Mittelstädt et al., 2019). The present results suggest that the relative activation of different task goals can be reactively biased by temporal differences when people are not instructed to randomly select tasks as in previous studies (Arrington & Weaver, 2015; Arrington, 2008). Thus, while the external biases in previous studies could reflect a failure to internally select a task based on the randomness instruction and/or that participants used external factors to aid their randomness choices, the present study favors an interpretation in terms of flexible reactive task-choice adjustments during a trial. Specifically, to our knowledge, this is the first study that provides direct evidence for the idea that people can control the strength of external influences in driving voluntary task choices. This extends previous findings of reactive control adjustments in regulating (multi-) task performance (e.g., Jiang et al., 2018; Leonhard et al., 2011; Miller et al., 2009; Mittelstädt & Miller, 2017). Thus, effects of environmental inputs on task choices appear to reflect adaptive background monitoring that promotes flexible behavior rather than automatic triggering of task goals (e.g., Goschke, 2003; Goschke & Dreisbach, 2008).

An additional argument in support of the notion that external influences promote flexible behavior rather than stable behavior could be the finding that the interaction between trial-specific and frequency-SOA, which serves as an indicator of a reactive control strategy, exhibited a stronger manifestation when participants chose to switch tasks compared to when they repeated tasks (see Appendix B^6^). Interestingly, in additional (not preregistered) analyses using switch percentages as the dependent measure instead of S1-task percentages, we observed an overall increase in participants' switching behavior in blocks where they anticipated a higher number of long SOAs (as well as larger switch rates with longer trial-specific SOAs).^7^ This finding suggests that participants indeed adopted a more flexible switching mode between the two tasks based on the proportion of information availabilities, which extends previous research that demonstrated an increased preference for task switching when the proportion of forced- task choices was higher (e.g., Mendl & Dreisbach, 2022). In future studies, it would be interesting to investigate whether the adoption of a more flexible mode in the present study, based on environmental availabilities, is generally more abstract or specific to the tasks involved (e.g., by implementing SOA manipulations in two tasks and examining potential transfer effects on task choices in intermixed diagnostic tasks without any SOA-based manipulations).

On a theoretical level, one could further speculate that people monitor the current availability of multiple potential task-specific pieces of information in parallel as a function of the overall task requirements before selecting a task goal for deliberate task processing. From a functional perspective, this flexibility allows individuals to consider both internal (cognitive) and external (environmental) costs and benefits when evaluating the expected effort required to complete a task relative to the expected payoff, and to adjust their task-selection behavior accordingly (e.g., Mittelstädt et al., 2018b; Shenhav et al., 2021). In the present study, participants may have been motivated to minimize trial duration and optimize their behavior accordingly. As a result, the findings are consistent with previous research employing the self-organized task switching paradigm (e.g., Mittelstädt et al., 2019, 2021), where stimulus availability was contingent upon task choice and allowed participants to anticipate task-specific availabilities prior to each trial. Notably, in these earlier studies, proactive task-choice processes likely played a significant role in adapting task choices based on the upcoming task-specific SOA. In contrast, in the present study, the interactive effects observed between the trial-specific and block-wise frequency-SOA are primarily interpreted as reflecting a reactive control strategy, given that participants could not anticipate the specific task-associated availabilities in advance. However, the idea that performance optimization may drive voluntary behavior in the present study does not negate the possibility of demand avoidance also impacting behavior. It is plausible to argue that the activation of task sets based on the presentation of task-relevant stimuli (e.g., Arrington et al., 2010; Koch & Allport, 2006) requires additional inhibitory processes to prevent responding to the first-presented task stimulus.

Finally, on a practical level, the present findings suggest it is important to remove distracting information related to a currently not-relevant task goal to enable stable and focused behavior on a single task when we are not fully capable of offloading the distractor task goal from working memory and have limited time to internally prioritize a task.

## Figures and Tables

**Fig. 3:**
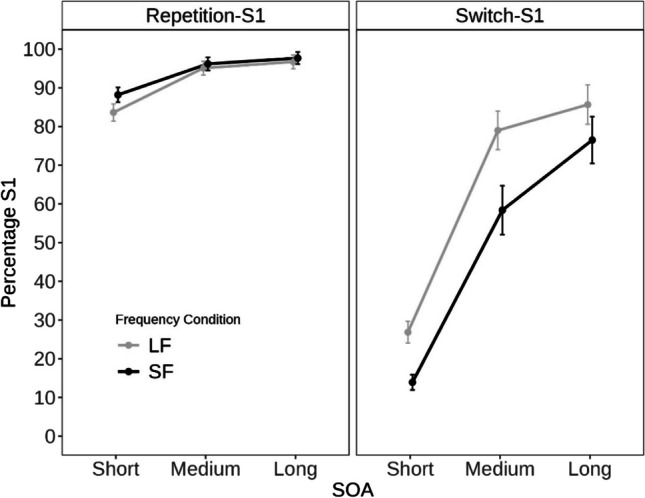
Percentage of trials in which participants selected the task related to the first-presented stimulus as a function of the trial-specific stimulus-onset asynchrony (SOA) in trial n, the frequency of SOAs (short SOA frequent (SF), long SOA frequent (LF)) in this block and whether S1 was associated with switching (switch-S1) or repeating tasks (repetition-S1)

**Fig. 4:**
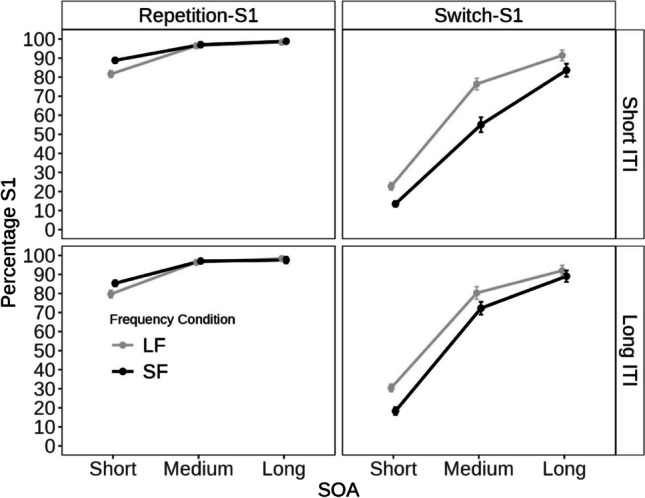
Percentage of trials in which participants selected the task related to the first-presented stimulus as a function of the trial-specific stimulus-onset asynchrony (SOA) in trial n, the frequency of SOAs (short SOA frequent (SF), long SOA frequent (LF)) in this block, the intertrial interval (ITI, short vs. long) and whether S1 was associated with switching (switch-S1) or repeating tasks (repetition-S1)

## Appendix A

In this appendix, we explore whether the critical findings reported in the main text using ANOVAs can be replicated when analyzing task-choice behavior (measured by S1-task percentages) using generalized mixed-effect (GME) modeling.

**Experiment 1.** Table 2 presents the corresponding results of the S1-task percentages GME analysis. The model included trial-specific SOA (short, medium, long), frequency-condition (short frequent (SF), long frequent (LF)), and the interaction between trial-specific SOA and frequency-SOA as fixed effects, with random intercepts for participants^8^. Similar to the results reported in the main, the interaction was found to be significant. Additionally, follow-up GME analyses for the two different trial-specific SOAs yielded similar results, indicating that the interaction was significant in the GME comparing short and medium trial-specific SOA (*χ2* (1) *=* 43.33*, p* <.001) as well as in the GME comparing medium and long trial-specific SOA (*χ2* (1) *= 67.75, p* <.001). The interaction was also significant when comparing short and long trial-specific SOAs (*χ2* (1) *=* 13.24*, p* <.001).

**Experiment 2.** Table 3 presents the corresponding results of the S1-task percentages obtained from the GME model analysis. The model included trial-specific SOA (short, medium), frequency-SOA condition (short frequent (SF), long frequent (LF)), ITI (short-ITI, long ITI) and the corresponding interactions as fixed effects, with participants treated as random effects.

Similar to the results reported in the main text using ANOVA, the two-way interaction between trial-specific SOA and frequency-SOA condition was also significant (see Table 3). Furthermore, consistent with the results reported in the main text, separate GMEs for each ITI condition, revealed that this interaction was significant for the short-ITI (*χ2* (2) *=* 48.72*, p* <.001) but not for the long-ITI (*χ2* (2) *= 1.96, p* = .376).

## Appendix B

In this appendix, we report additional analyses on task-choice behavior (as measured with S1-task percentages) while additionally considering whether S1 was associated with a switch or repetition.

**Experiment 1**

We first computed the S1-task percentage as a function of trial-specific SOA (short, medium, long), frequency-SOA condition (SF, LF) and S1-transition type (switch-S1 vs. repetition S1). As can be seen in Fig. 3 there was a strong repetition bias as reflected in a larger preference to select S1 for repetition-S1 than switch-S1.

A 3 × 2 × 2 ANOVA revealed that all effects were significant (all *p*s < .015)–including the three-way interaction (*p* = .001; η_p_^2^ = .14). We conducted separate ANOVAs for switch-S1 and repetition-S1 with the factors of SOA (short, medium, long), and frequency-SOA condition (SF, LF). For switch-S1, the two main effects as well as the 2 × 3 interaction was significant (all *p*s < .008; all η_p_^2^ > .15)^9^. For repetition-S1, the main effect of SOA was significant (*p* < .001; η_p_^2^ > .60) and the 2x3 interaction was not significant (*p* = .097; η_p_^2^ = .08).

**Experiment 2**

In additional analyses, we again considered whether S1 was associated with switching versus repeating tasks (see Fig. 4).

Similar to the analyses reported in the main text, we focused on the trial-specific SOA = short and trial-specific SOA = medium trials. Specifically, we conducted separate 2 × 2 × 2 ANOVAs for switch-S1 and repetition-S1 of trial-specific SOA (short, medium), frequency-SOA condition (SF, LF) and ITI (short-ITI, long-ITI) on the S1-task percentages.

For switch-S1, all three main effects were significant (all *p*s < .001 and all η_p_^2^ > .48). The two-way interaction between SOA and ITI was also significant, *p* = .025, η_p_^2^ = .09, reflecting a larger trial-specific SOA effect on S1-task percentages at long-ITI (76.2–24.3%) than at short-ITI (65.7–18.1%). The two-way interaction between frequency-SOA condition and ITI was also significant, *p* = .004, η_p_^2^ = .15, reflecting a larger frequency-SOA effect on S1-task percentages at short-ITI (49.6–34.3%) than at long-ITI (55.3–45.2%). Critically, the three-way interaction was also significant, *p* < .001, η_p_^2^ = .25. Separate 2 × 2 ANOVAs were conducted for each ITI condition. For the short-ITI condition, the two main effects as well as the interaction was significant (all *p*s < .002 and all η_p_^2^ > .18). For the long-ITI condition, the two main effects (all *p*s < .001 and all η_p_^2^ > .37), but not the interaction (*p* = .164 and η_p_^2^ = .04) were significant. As can be seen in Fig. in Fig. 4, only for short-ITI (but not long-ITI), there was a larger difference in S1-task percentages between short- and medium-SOA in LF than SF blocks.

For repetition-S1, all three main effects were significant (all *p*s < .024 and all η_p_^2^ > .09). The two-way interaction between trial-specific SOA and frequency-SOA condition was also significant, *p* < .001, η_p_^2^ = .25, reflecting a larger SOA effect (i.e., S1-task percentages in medium versus short SOA trials) on S1-task percentages in LF (96.4–80.6%) than in SF blocks (97.0–87.1%). Furthermore, the two-way interaction between SOA and ITI was also significant, *p* = .026, η_p_^2^ = .09, reflecting a larger trial-specific SOA effect on S1-task percentages at long-ITI (96.7%–82.5%) than at short-ITI (96.8–85.2%). The three-way interaction was not significant, (*p* = .495, *η*_*p*_^*2*^ = .01). For completeness, separate 2 × 2 ANOVAs were still conducted for each ITI condition. At short-ITI, the two main effects as well as the interaction was significant (all *p*s < .002 and all η_p_^2^ > .20). At long-ITI, the two main effects and the interaction were also significant (all *p*s < .012 and all η_p_^2^ > .11). As can be seen in Fig. 4, for both short-ITI and long-ITI, there was a larger trial-specific SOA effect on S1-task percentages in the LF than SF blocks.

## Appendix C

In this Appendix, we report additional analyses for Experiment 2 including the long-SOA condition. The 3 × 2 × 2 ANOVA with the within-subject factor of current-SOA (short, medium, long), proportion-SOA (long-wait, short-wait) and ITI (short-ITI, long-ITI) on the S1-task proportion revealed a significant three-way interaction was also significant, *p* < .001, η_p_^2^ = .16.

The 2 × 2 × 2 ANOVA with the within-subject factor of current-SOA (*medium, long*), proportion-SOA (long-wait, short-wait) and ITI (short-ITI, long-ITI) on the S1-task proportion revealed that the three-way interaction (*p* = .031, η_p_^2^ = .09) and all other effects were significant (all *p*s < .002, η_p_^2^ > .19). Separate 2 × 2 ANOVAs with the factors of current-SOA (medium, long), proportion-SOA (long-wait, short-wait) were conducted for each ITI condition. For the short-ITI condition, the two main effects as well as the interaction was significant (all *p*s < .002 and all η_p_^2^ > .20). For the long-ITI condition, the two main effects (all *p*s < .018 and all η_p_^2^ > .10), but not the interaction (*p* = .112 and η_p_^2^ < .06) was significant.

The 2 × 2 × 2 ANOVA with the within-subject factor of current-SOA (*short, long*), proportion-SOA (long-wait, short-wait) and ITI (short-ITI, long-ITI) on the S1-task proportion revealed that the three-way interaction (*p* = .007, η_p_^2^ = .14) and all main effects were significant (all *p*s < .004, η_p_^2^ > .13), but not the two-way interactions (all *p*s > .286, η_p_^2^ < .03). Separate 2 × 2 ANOVAs with the factors of current-SOA (short, long), proportion-SOA (long-wait, short-wait) were conducted for each ITI condition. For the short-ITI condition, the two main effects (all *p*s < .037 and all η_p_^2^ > .07), but not the interaction (*p* = .105 and η_p_^2^ = .06) was significant. For the long-ITI condition, the two main effects (all *p*s < .001 and all η_p_^2^ > .22) were significant, whereas the interaction was not significant (*p* = .079 and η_p_^2^ = .06).

**Table 2:** Generalized mixed-effects model analysis of Experiment 1 reported with S1-task percentages as dependent variable

Effect	*χ2*	*p*
Trial-specific SOA	2474.85	<.001
Frequency-SOA	107.69	<.001
Trial-specific SOA:Frequency-SOA	49.29	<.001

***Note.*** Trial-specific SOA (short, medium, long), frequency-SOA condition (short frequent (SF), long frequent (LF)) and the interaction between SOA and frequency-SOA were included as fixed effects, while participants were included as random effects across all analyses (model specification: dv ~ Trial-specific-SOA*Frequency-SOA + [1 | Participant]). Analyses were implemented using the lme4 package (Bates et al., 2015) via the mixed function within the afex package (Singmann et al., 2021). The family argument was “binomial” with a logit link function, with p-values provided by likelihood-ratio tests

**Table 3:** Generalized mixed-effects model analysis of Experiment 2 reported with S1-task percentages as dependent variable

Effect	*χ2*	*p*
Trial-specific SOA	6059.02	< .001
Frequency-SOA	95.12	< .001
ITI	36.04	< .001
Trial-specific-SOA:Frequency-SOA	33.46	< .001
Trial-specific-SOA:ITI	13.49	.001
ITI:Frequency-SOA	4.20	.041
Trial-specific-SOA:Frequency-SOA:ITI	14.14	< .001

***Note.*** Trial-specific SOA (short, medium), frequency-SOA condition (short frequent (SF), long frequent (LF)), ITI (short-ITI, long-ITI), the three 2-way interaction and the 3-way interaction were included as fixed effects, while participants were included as random effects across all analyses (model specification: dv ~ Trial-specific-SOA*Frequency-SOA*ITI + [1 | Participant]). Analyses were implemented using the lme4 package (Bates et al., 2015) via the mixed function within the afex package (Singmann et al., 2021). The family argument was “binomial” with a logit link function, with p-values provided by likelihood-ratio tests

## Footnotes

1 Preregistration is available via the Open Science Framework (OSF) at https://osf.io/u54fb. The sample size was somewhat arbitrarily yet conservatively set (based on effect size estimates from pilot data) in order to compensate for potential drop-outs. A power analysis using the R-package Superpower (Lakens & Caldwell, 2021) indicated that with the actual sample size of 31 participants we would have over 80% power with a significance level of 5% to detect an effect of at least η_p_^2^ = 0.22 for the 2 × 2 interaction of frequency-SOA (SF, LF) and trial-specific SOA (short, medium).

2 Specifically, after our data-trimming procedure, we examined whether participants followed any consistent global task-choice strategies in free-choice trials (i.e., always selecting the same task or always selecting the same or different task that was performed in the previous trial) that may overshadow any potential effects of the central SOA manipulation. Specifically, we first checked the individual switch rates and we excluded the data of nine participants who repeated tasks in > 95% of trials. We reasoned that these participants predetermined their task choice before the actual experiment (e.g., "I will consistently choose the number task"), suggesting a lack of adaptability to the task environment. However, it is noteworthy that the results in the two experiments remained similar even when including these participants. Therefore, the decision to exclude these participants does not impact the overall conclusions. Data of one additional participant was excluded due to showing a strong preference for selecting the number tasks (i.e., > 95% of number task choices).

3 As shown in Appendix A, both in this experiment and the next one, very similar results were obtained when employing generalized mixed-effect modeling analyses.

4 To investigate this 2 × 3 interaction in more detail, separate frequency-SOA × trial-specific SOA ANOVAs were carried out for the two different trial-specific SOAs. The interaction was significant in the ANOVA comparing short and medium trial-specific SOA (*p* = .011, η_p_^2^ = .21) and the ANOVA comparing medium and long trial-specific SOA (*p* < .001, η_p_^2^ = .44), but not in the ANOVA comparing short and long trial-specific SOAs (*p* = .679, η_p_^2^ = .01).

5 Unfortunately, we realized after data collection that we had forgot to upload the preregistration document. However, we have uploaded a pdf file of the preregistration with a saving date before data collection started (see https://osf.io/hb3tj).

6 As we have previously mentioned, caution must be exercised when interpreting the absence or smaller magnitude of the interaction on S1-task percentages under repetition-S1 and switch-S1 conditions. Specifically, it is important to note that the consistently high S1-task percentages with repetition stimuli result in limited variability and reduced sensitivity to detect potential interactions (see Loftus, 1978). In future studies, it may be beneficial to explore methods to minimize switching behavior in order to mitigate potential ceiling effects. This could be achieved by implementing practices that involve pre-experimental training in task switching or utilizing tasks that inherently facilitate task switching.

7 Specifically, we conducted a 3 × 2 ANOVA with trial-specific SOA (short, medium, long) and block-wise frequency-SOA (SF, LF) on switch rates for both Experiment 1 (SF blocks: SOA_short_ = 12.9%; SOA_medium_ = 32.4%, SOA_long_ = 39.5%; LF blocks: SOA_short_ = 22.5%; SOA_medium_ = 44.2%, SOA_long_ = 45.0%) and Experiment 2 (collapsed across ITI; SF blocks: SOA_short_ = 14.5%; SOA_medium_ = 32.8%, SOA_long_ = 44.0%; LF blocks: SOA_short_ = 22.9%; SOA_medium_ = 41.3%, SOA_long_ = 46.5%). These analyses revealed that in both experiments the main effects and the interaction was significant (all *p*s < .025; all η_p_^2^ > .12). Note that these analyses do not take into account whether participants selected the task associated with the first stimulus or the second stimulus.
